# A flexible Bayesian hierarchical model of preterm birth risk among US Hispanic subgroups in relation to maternal nativity and education

**DOI:** 10.1186/1471-2288-11-51

**Published:** 2011-04-19

**Authors:** Jay S Kaufman, Richard F MacLehose, Elizabeth A Torrone, David A Savitz

**Affiliations:** 1 Department of Epidemiology, Biostatistics and Occupational Health, McGill University, Montreal, QC, Canada; 2 Departments of Epidemiology and Biostatistics, University of Minnesota, Minneapolis, MN, USA; 3 Department of Epidemiology, University of North Carolina at Chapel Hill, NC, USA; 4 Department of Community Health, Brown University, Providence, RI, USA

## Abstract

**Background:**

Previous research has documented heterogeneity in the effects of maternal education on adverse birth outcomes by nativity and Hispanic subgroup in the United States. In this article, we considered the risk of preterm birth (PTB) using 9 years of vital statistics birth data from New York City. We employed finer categorizations of exposure than used previously and estimated the risk dose-response across the range of education by nativity and ethnicity.

**Methods:**

Using Bayesian random effects logistic regression models with restricted quadratic spline terms for years of completed maternal education, we calculated and plotted the estimated posterior probabilities of PTB (gestational age < 37 weeks) for each year of education by ethnic and nativity subgroups adjusted for only maternal age, as well as with more extensive covariate adjustments. We then estimated the posterior risk difference between native and foreign born mothers by ethnicity over the continuous range of education exposures.

**Results:**

The risk of PTB varied substantially by education, nativity and ethnicity. Native born groups showed higher absolute risk of PTB and declining risk associated with higher levels of education beyond about 10 years, as did foreign-born Puerto Ricans. For most other foreign born groups, however, risk of PTB was flatter across the education range. For Mexicans, Central Americans, Dominicans, South Americans and "Others", the protective effect of foreign birth diminished progressively across the educational range. Only for Puerto Ricans was there no nativity advantage for the foreign born, although small numbers of foreign born Cubans limited precision of estimates for that group.

**Conclusions:**

Using flexible Bayesian regression models with random effects allowed us to estimate absolute risks without strong modeling assumptions. Risk comparisons for any sub-groups at any exposure level were simple to calculate. Shrinkage of posterior estimates through the use of random effects allowed for finer categorization of exposures without restricting joint effects to follow a fixed parametric scale. Although foreign born Hispanic women with the least education appeared to generally have low risk, this seems likely to be a marker for unmeasured environmental and behavioral factors, rather than a causally protective effect of low education itself.

## Background

A great deal of research in reproductive and social epidemiology has focused on the "Hispanic Paradox", in which Hispanic women of low socioeconomic status (SES) in the United States (US) have better than expected birth outcomes, compared to other similarly disadvantaged groups, such as African-Americans [1]. Some authors have suggested that acculturation is a key modifier of the apparently protective effect of Hispanic ethnicity [2]. For example, Acevedo-Garcia and colleagues observed that the well known protective effect of higher socioeconomic position of the mother was more modest for foreign born Hispanics than for the native born [3]. But the "Hispanic" label is North American construction that masks considerable variability between groups [4]. Acevedo-Garcia et al therefore provided a systematic investigation of the interaction between nativity, maternal education (as a marker for SES) and Hispanic subgroup on low birth weight (LBW). In 2002 US Natality Detail data, with a sample size of over 630,000 singleton births to US Hispanic women, the authors used logistic regression with interaction terms to document variation in the association between nativity and LBW by Hispanic subgroup, and an interaction between nativity and education for some subgroups. They reported that when stratified by ethnic subgroups and nativity, the "Hispanic Paradox" is apparent only for foreign-born Mexicans and Central/South Americans. For foreign-born Puerto Ricans and Cubans, increasing education was associated with decreased LBW risk [5].

We extend the previous research in several ways using a dataset from New York City. Although our sample size is smaller than that used by Acevedo-Garcia et al, by using Bayesian random effects regression models we are able to employ finer categorizations of both education and Hispanic subgroups to depict additional heterogeneity in risk of adverse birth outcomes and variations in the degree to which advancing education is associated with reduced risk across Hispanic subgroups, all on the absolute scale. We propose that the use of a single geographic region is advantageous in contrast with national data in order to avoid confounding by many unmeasured regional differences, such as the historically unique context of Cuban-Americans in South Florida or Mexican-Americans in the Southwest.

The absolute scale is a more useful contrast to make in a public health context because it directly represents the number of attributable cases, and therefore the actual public health burden [6]. The use of each ethnic group as its own reference when constructing odds ratio estimates make it is impossible for the reader to know whether one group has higher baseline risk than another and how variation in baseline risks affects the pattern of odds ratios across groups. A Bayesian modeling approach offers a relatively simple way to obtain effects on the absolute scale along with measures of precision, a task that would be very challenging using frequentist approaches given the use of random effects in a non-linear model like logistic regression. This paper therefore serves to demonstrate the advantages of this analytic approach in terms of fitting the models and representing the output using simple graphs that clearly represent the estimated dose-response function in various groups.

Low birthweight, defined as births of less than <2500 g, has been widely used as a convenient measure of an adverse birth outcome. However, LBW encompasses infants with a mix of underlying pathologies: those who are growing normally but are born too early (i.e. preterm), and those that are full term but small from stunted fetal growth (i.e. intra-uterine growth retardation). Therefore, we focus in the current report on preterm birth (PTB) as a more etiologically distinct outcome [7], especially as recent studies have demonstrated the importance of prematurity on morbidity and mortality throughout the lifecourse [8,9].

## Methods

### Data description

We used publicly available vital statistics birth data for 1995 to 2003 from the NYC Department of Health and Mental Hygiene. To remain consistent with the previous analysis by Acevedo-Garcia et al [5], and to allow completed years of education to have a meaningful interpretation, we excluded births to women under age 20. Following common practice, we also restricted to singleton births because of the markedly distinct patterns of fetal growth and gestational age in non-singleton pregnancies. We included all women self-identifying as Hispanic or Latino. However, we categorized these more finely than in previous reports, and we included births to women of "other or unknown" Hispanic origin, classifying them as their own subgroup.

### Variables

Preterm birth was defined as delivery for any reason prior to 37 completed weeks of gestation using the clinical estimate. Maternal education was based on self-reported years of completed schooling. Although previous authors categorized years of education broadly, we included the exact number of years reported, up to a maximum of 17 years, using a flexible regression model. Nativity was dichotomized as foreign-born or native-born, where for Puerto Rican women this corresponded to the distinction between being born on the island of Puerto Rico ("foreign born") and being born in the mainland United States ("native born"). Hispanic subgroups were based on maternal self-reported ancestry and ethnicity and categorized as: Mexican, Puerto Rican, Cuban, Dominican, Central American (Belize, Costa Rica, El Salvador, Guatemala, Honduras, Nicaragua, Panama), South American (Argentina, Bolivia, Brazil, Chile, Columbia, Ecuador, Paraguay, Peru, Uruguay, Venezuela) and Other/Unknown.

Models were adjusted either for age alone, or for covariates selected to replicate the previously published analysis. These included: prenatal care, defined by the Kessner Index as adequate, intermediate or inadequate care [10], sex of child, maternal age (categorized as 20-24, 25-29, 30-34, 35-39, 40+), previous live birth (categorized as 0, 1-4, 5+) and dichotomous measures of: tobacco use during pregnancy, alcohol use pregnancy and medical risk factors (anemia, pregnancy-associated hypertension, diabetes, uterine bleeding, preeclampsia, eclampsia, placenta previa, and placental abruption).

### Statistical analysis

We estimate the risk of PTB by years of maternal education, nativity and Hispanic subgroup using a Bayesian random-effects logistic regression model with restricted quadratic splines and knot locations placed at 8, 11, and 13 years. The form of the model is:(1)

where Y_ijk _is defined as the binary PTB outcome for the i^th ^woman (for i = 1...n_jk_) in the j^th ^ethnic group (for j = 1...7) and the k^th ^nativity group (for k = 0,1), with adjustment for m baseline covariates, x, by means of estimated coefficients β_m_. The terms q_1ijk _to q_3ijk _are restricted quadratic spline terms, where the qualifier "restricted" implies a linear dose response relationship in the region less than 8 years and the region greater than 13 years of education. The α_1jk _... α_3jk _coefficients determine what the education dose-response curve looks like for the infants of mothers in ethnic group j and nativity class k. The α coefficients are specified as random effects so that the education functions may borrow information between ethnic groups of the same nativity class (but not across different nativity classes). In particular, we specify:(2)

Each of the α_gjk_; g = 1...3, j = 1...7, k = 0,1 is shrunk toward the group mean for that particular nativity class [11]. That is, each of the spline coefficients borrows strength from the spline coefficient of other ethnic groups within that nativity class. The amount of information borrowed between ethnic groups (and therefore the amount of shrinkage), is determined by the precision term, τ. A large value of τ_3_, for example, indicates that the spline coefficients for foreign born mothers will borrow a larger amount of information from one another.

The Bayesian approach requires priors to be specified for all unknown parameters. The specifications for these priors were selected to be relatively uninformative since little data exists to specify informative priors on the spline coefficients in our model. Further, given the large number of observations prior information is unlikely to have any substantial impact on the results. The prior mean parameters,  were assumed to follow independent normal distributions with mean 0 and variance 1. The inverses of the variance terms τ_0 _...τ_3 _were assumed to be independently gamma distributed with shape and rate parameters 0.1 and 0.1. Finally, the prior distribution of the remaining coefficients in expression [1], β_m_, were assumed independent and identically normally distributed with mean 0 and variance 10.

Models were fit using WinBUGS version 1.4.3 [12]. To facilitate convergence, we centered spline variables in the model (Additional file 1). Markov chain Monte Carlo (MCMC) algorithms were run for 1,000,000 iterations following a 10,000 iteration burn-in. We retained every 10^th ^iteration to reduce autocorrelation between samples as well as for analytic tractability. Convergence was assessed by visual examination of traceplots (Additional file 2: appendix figure A6). Analyses were repeated with the Markov chains started from different locations to help ensure convergence to a stable posterior distribution. Finally, we fit additional models to test the sensitivity of our results to different prior specifications. In particular, we ensured that results were consistent with the specification of more diffuse distributions on prior parameters.

## Results

Over the eight year period there were 990,597 singleton births to women ≥20 years of age. We restricted the analysis to women self-identified as Hispanic or Latina (n = 365,139). We excluded from the analysis observations missing birthweight (n = 81), nativity status (n = 5,962), ethnic ancestry (n = 9,416) or education (n = 15,254), for a cumulative exclusion of n = 26,550 (2.7%). We also excluded all observations that were missing any covariate when fitting the fully adjusted model, although no covariate was missing more than 1.2% with the exception of prenatal care (11.4%), which left a final sample size of 258,680 for the fully adjusted analyses.

Similar to findings in previous reports, demographics and maternal education varied by nativity and Hispanic subgroup (Table 1). The risks of adverse birth outcome measures, including PTB, were higher among US born women than foreign born women, with the exception of women from Puerto Rico and South America. Foreign-born women also reported substantially fewer years of completed education. Mean years of schooling completed for foreign born Mexican women, for example, was 8.7. In contrast, no US-born ethnic group had a mean level of schooling less than 12 years. Because educational attainments less than 8 years were exceedingly rare in women born in the US, estimates below this level were not plotted for the native born group.

**Table 1:** Descriptive statistics of singleton births among mothers ≥20 years old by Hispanic subgroup and nativity: New York City, 1995-2003

	**All Hispanic/Latino**		**Mexican**		**Puerto Rican**		**Cuban**		**Dominican Republic**		**Central American**^**1**^		**South American**^**2**^		**Non-specific Hispanic**^**3**^	
	**US born**	**Foreign born**	**US born**	**Foreign born**	**US born**	**Foreign born**	**US born**	**Foreign born**	**US born**	**Foreign born**	**US born**	**Foreign born**	**US born**	**Foreign born**	**US born**	**Foreign born**
	
Population (nativity %)	82,427 (31.8)	177,169 (68.2)	1,073 (2.7)	38,416 (97.3)	55,751 (75.5)	18,062 (24.5)	1,360 (60.0)	906 (40.0)	8,686 (13.0)	57,943 (87.0)	1,687 (8.6)	17,911 (91.4)	3,845 (9.8)	35,429 (90.2)	10,025 (54.1)	8,502 (45.9)
Preterm (%)	8.83	6.75	6.34	6.32	9.37	9.72	8.38	8.28	7.61	6.82	7.17	7.00	7.13	5.70	8.16	5.65
Low birthweight (%)	7.26	5.19	5.78	4.52	7.87	8.01	6.32	5.52	6.33	5.35	5.99	5.65	4.14	4.24	6.39	4.13
Term low birthweight^4 ^(%)	2.93	2.09	2.99	1.93	3.26	3.26	2.33	2.05	2.39	2.08	2.30	2.37	1.54	1.68	2.30	1.68
Maternal age 35+ years (%)	10.95	16.75	14.26	7.34	12.42	18.74	22.06	42.60	4.18	17.11	6.94	18.31	7.62	23.74	8.74	17.45
Maternal Education																
Mean Years (sd)	12.6 (2.2)	11.1 (3.3)	12.9 (2.9)	8.7 (3.4)	12.3 (2.1)	11.9 (2.4)	14.3 (2.2)	14.2 (2.5)	13.2 (2.1)	12.0 (2.7)	13.6 (2.1)	10.8 (3.4)	13.9 (2.1)	12.0 (3.2)	12.4 (2.0)	11.6 (2.8)
Adequate prenatal care (%)	54.49	50.76	55.36	48.03	53.83	50.80	65.59	65.23	57.68	51.69	58.15	51.89	59.90	51.39	51.89	50.12
Smoking during pregnancy (%)	6.32	1.40	2.06	0.18	7.66	7.18	3.54	2.65	2.62	1.23	2.26	0.61	1.67	0.59	5.35	0.84
Drinking during pregnancy (%)	0.30	0.11	0.28	0.03	0.35	0.35	0.37	0.33	0.17	0.13	0.18	0.11	0.08	0.08	0.22	0.07
Missing smoking/Drinking (%)	0.5	0.3	0.4	0.2	0.5	0.6	0.5	0.3	0.2	0.3	0.2	0.3	0.2	0.3	0.9	0.1

1. Counties included: Belize, Costa Rica, El Salvador, Guatemala, Honduras, Nicaragua, Panama

2. Countries included: Argentina, Bolivia, Brazil, Chile, Columbia, Ecuador, Paraguay, Peru, Uruguay, Venezuela

3. Nationality listed as "Hispanic"

4. Proportions among full term (37+ weeks) gestations only

**Figure 1:**
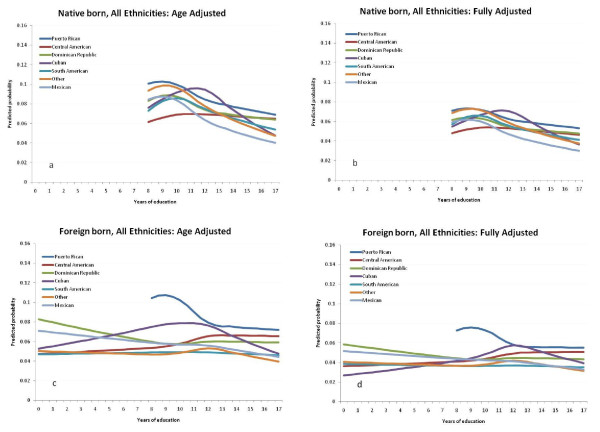
**Age-adjusted and fully-adjusted risks of low birthweight (PTB) across maternal education levels by Hispanic subgroups among native and foreign born women ≥20 years old, New York City, 1995-2003**. Panel a: native born, age-adjusted; Panel b: native born, full-adjusted; Panel c: foreign born, age-adjusted; Panel d, foreign-born, fully-adjusted.

Figure 1 shows the age-adjusted and fully adjusted absolute risks of PTB for all 7 ethnic groups. Risk estimates are computed for mothers 20-24 years in the age-adjusted models. In the fully adjusted models, the referent group for adjustment is defined by the lowest risk covariate pattern: mothers 20-24 years old, without hypertension, preeclampsia, eclampsia, uterine bleeding, placental abruption, placenta previa, diabetes, who did not use tobacco or alcohol while pregnant, received adequate prenatal care, had 1-4 previous live births and whose infants were female, Native born women are shown in the two upper panels (**a **and **b**) and foreign born women in the two lower panels (**c **and **d**) of Figure 1. Individual ethnic-group plots with 95% posterior intervals to represent precision of these estimates are shown in appendix figures A1-A2 (Additional file 2). Puerto Rican and Cuban women show consistently elevated risk compared to other groups and strong protective effects of higher education. Foreign born women derive less benefit from advanced education, and often show much lower absolute risk at low levels of schooling, with most groups having maximum risk of PTB around 10 to 12 years of schooling.

**Figure 2:**
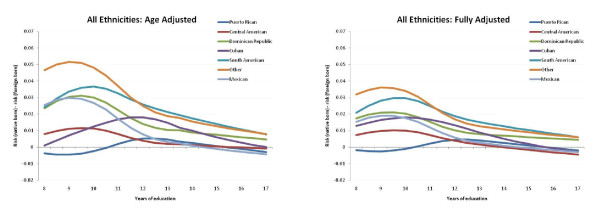
**Age-Adjusted (left panel) and fully-adjusted (right panel) risk differences (RD) of preterm birth (PTB) across maternal education levels by Hispanic subgroups**. The RD contrast is risk in native born minus risk in foreign born mothers, for women ≥20 years old, New York City, 1995-2003.

Figure 2 shows the estimated age-adjusted risk difference (RD) contrasting native and foreign born mothers at each year of completed education starting at 8 years (the effective minimum for native born women). The left panel contains the age-adjusted effect estimates and the right panel contains the fully-adjusted effect estimates. Individual ethnic-group RD plots with 95% posterior intervals to represent precision of these estimates are shown are shown in appendix figures A3-A4 (Additional file 2). Once again, the Cuban and Puerto Rican groups have a distinct pattern in which nativity matters little across the range of educational accomplishments. The other groups, however, show a marked effect of nativity at low educational levels (i.e. more favorable outcomes for less educated foreign-born than US-born mothers) and a monotonic decline in the protective impact of nativity with increasing educational level, such that nativity is completely inconsequential for those with highest levels of education in all groups.

## Discussion

Using vital statistics birth records from New York City, we have extended previous investigations of the "Hispanic Paradox", but with a novel statistical methodology that allowed for numerous improvements. The use of Bayesian hierarchical modeling allowed for the estimation and graphing of all effects on the absolute scale, which has more direct public health relevance and allows for direct comparison between groups without having to specify a referent group for the contrast parameter. The methodology also permits easy calculation of posterior intervals, whereas the calculation of variances for posterior probability estimates from multilevel logistic models in a frequentist setting would have been enormously difficult. Moreover, the shrinkage accomplished with random coefficient terms allowed for flexible modeling over relatively fine categorizations of education and ethnicity in order to produce more specific patterns than reported previously, and with the extent of shrinkage determined by the data. Finally, we reported effects for a more pathologically specific outcome, preterm birth, which represents a more homogeneous etiology (truncated gestational age) than the composite outcome of low birthweight (LBW) that has often been reported [3,5].

We also highlight the age-adjusted rather than the fully-adjusted estimates because we argue that these are particularly important for understanding disease burden in populations, since the real situation of these women and their pregnancies is more readily revealed in the age-adjusted values. Moreover, we would argue that for etiologic inference, the covariate-adjusted estimates may be less helpful because the covariates are, except for age, arguably consequences of the primary exposures: nativity and ethnicity [13]. Furthermore, as shown in appendix figure A5 (Additional file 2), the adjustment actually makes little practical difference to the effect measures in this instance.

Random-effects regression models have the advantage of shrinking group-specific estimates toward the adjusted education-category mean risks, which guarantees a reduced mean square error for the ensemble of results [12]. This implies that coefficient estimates based on sparse categories in the data will "borrow strength" (i.e., shrink coefficients toward a common prior mean) from their neighbors in order to avoid erratic estimates and that estimated values are "smoothed" in order to better recognize the underlying patterns in the data [14]. The models we employed considered the 7 ethnicity groups to be exchangeable at each nativity stratum and year of achieved education, conditional on the modeled covariates. Although this approach can be conservative, since it biases truly dissimilar values toward the group mean [15], it nonetheless allowed us to estimate risks for finer categorizations of both ethnicity and education level. Further, the extent of borrowing in our model is determined by the prior precision terms τ, which are estimated, in part, from the data. This adds a level of robustness to our model: when the data reflect greater heterogeneity between groups, the precision term will decrease to ensure little borrowing of information [16].

Previous analyses defined low education categorically as 0-11 years, and then reported a monotonic decrease in risk of adverse outcomes with increasing education [3,5]. By using finer classification of education, particularly in the lower range, and flexible dose-response modeling, we show that the data provide some evidence against a monotonic relationship for many of the Hispanic subgroups in NYC. For example, foreign-born women of very low education (less than 8 years) are estimated to have similar risk to women completing secondary education in many of the groups. It is quite likely that education serves as a marker for acculturation, with women who report very low educational attainment having the most traditional cultural affiliation. This could provide a protective effect for birth outcomes through mechanisms such as diet, social support, and decreased risk behaviors such as substance abuse [17].

Additionally, plotting predicted probabilities rather than graphing relative measures of effect allowed us to look directly at risk dose-response across groups. For example, the plotting of predicted absolute risks not only reveals the substantially higher risk for Puerto Ricans, but also shows that for this group in particular, there is no apparent advantage for the "foreign born" (i.e., island-born) as there is for other ethnicities. This makes sense substantively, since all Puerto Ricans are US citizens, whether born in the mainland or on the island. Greater mobility is therefore possible between populations, with less migrant selectivity among those who relocate. The other group with little apparent effect of nativity was Cubans, although the very small number of foreign born Cuban mothers in New York City limited the precision of these estimates severely. Evidence that this is not a distinctively Caribbean phenomenon can be seen by contrasting Dominicans, whose risk profile looks very much like the other Central and South American groups.

It should be noted that because we display absolute risk estimates, the adjusted values shown must depend on the choice of level at which covariates are fixed in the analysis. We chose to set all covariates to values with lowest risk, meaning that the graphs displayed are the "best case" scenarios for each stratum defined by ethnicity and nativity; when additional risk factor are "switched on", the absolute risks will be greater than those shown.

## Conclusion

This paper uses novel statistical methods to extend previous findings concerning risk of adverse birth outcomes for Hispanic women as a function of ethnicity, nativity and years of completed education. We confirmed the previously published findings that the education gradient is much flatter for foreign-born women, with the exception of island-born Puerto Ricans. We went beyond previous research, however, to demonstrate that more substantively interpretable analyses are possible through the use of flexible hierarchical models. Finer categorizations help reveal evidence that the benefit associated with additional years of schooling may not be monotonic, but rather that women with the lowest levels of education show reduced risk compared to those with 8-11 years. Furthermore, by displaying Bayesian posterior risk estimates and their differences, rather than ratio measures of effect, we show heterogeneity between groups that is obscured when each group is used as its own referent. These results show a consistent disadvantage for Puerto Rican women at all education levels and for all outcomes. Furthermore, results that are adjusted for measured risk factors will also be more moderate than those that occur in the real world. If Puerto Ricans also have a more disadvantaged profile of these factors, their true risks will be even more disparate.

## Competing interests

The authors declare that they have no competing interests.

## Authors' contributions

JSK and EAT conceived the study, carried out initial analyses and wrote the manuscript. RFM developed the current analytic strategy and fit the models. DAS provided the data and subject matter expertise. All authors read and approved the final manuscript.

## Pre-publication history

The pre-publication history for this paper can be accessed here:

http://www.biomedcentral.com/1471-2288/11/51/prepub

## Supplementary Material

Additional file 1 **Annotated Winbugs Software Code**. Annotated Winbugs Software Code Click here for file

Additional file 2 **Appendix Figures**. Appendix Figure A1. Age-adjusted risks of preterm birth (PTB) by nativity, education and Hispanic subgroup, women ≥20 years old, New York City, 1995-2003. Appendix Figure A2. Fully-adjusted risks of preterm birth (PTB) by nativity, education and Hispanic subgroup, women ≥20 years old, New York City, 1995-2003. Appendix Figure A3. Age-adjusted risk differences for the effect of nativity on preterm birth (PTB) by education and Hispanic subgroup, women ≥20 years old, New York City, 1995-2003. Appendix Figure A4. Fully-adjusted risk differences for the effect of nativity on preterm birth (PTB) by education and Hispanic subgroup, women ≥20 years old, New York City, 1995-2003. Appendix Figure A5. Comparison of age-adjusted to fully-adjusted risk differences for the effect of nativity on preterm birth (PTB) by education and Hispanic subgroup, women ≥20 years old, New York City, 1995-2003. Appendix Figure A6. Trace plots for several selected terms in the fully adjusted model (α_10_, δ_10 _and τ_0_) for native born Mexican-American women. Click here for file
