# Supplementary material for: A flexible Bayesian hierarchical model of preterm birth risk among US Hispanic subgroups in relation to maternal nativity and education
Source: BMC Med Res Methodol. 2011 Apr 19;11:51. doi: 10.1186/1471-2288-11-51 (PMC3108375; doi:10.1186/1471-2288-11-51)
Supplement: Additional file 1 — Annotated Winbugs Software Code. Annotated Winbugs Software Code [file 1471-2288-11-51-S1.DOC]

Appendix: Annotated Winbugs Software Code

Data are collapsed into categories defined by maternal age and education to speed up processing. y[i] is the number of outcomes in the i^th^ category out of the n[i] people in that category. group[i] is an observed variable takes on values 1 to 14 for each ethnic/nativity combination. Groups 1 to 7 are native born and groups 8 to 14 are foreign born. Winbugs parameterizes the normal distribution in terms of the precision, rather than variance, which accounts for the difference between the parameterization here and eq. 2 in the paper.

model

{

for (i in 1:tot) {

y[i]~dbin(p[i],n[i])

logit(p[i])<-delta[group[i]] +a1s[group[i]]*cq1[i]+a2s[group[i]]*cq2[i]+a3s[group[i]]*cq3[i]

+igmage2[i]*xi2+igmage3[i]*xi3+igmage4[i]*xi4+igmage5[i]*xi5

}

for (j in 1: 7) {

delta[j]~dnorm(d0,taudelta1)

a1s[j]~dnorm(pma1,taualpha1)

a2s[j]~dnorm(pma2,taualpha1)

a3s[j]~dnorm(pma3,taualpha1)

}

for (j in 8: 14) {

delta[j]~dnorm(d1,taudelta2)

a1s[j]~dnorm(pmb1,taualpha2)

a2s[j]~dnorm(pmb2,taualpha2)

a3s[j]~dnorm(pmb3,taualpha2)

}

pma1~dnorm(0,1)

pma2~dnorm(0,1)

pma3~dnorm(0,1)

pmb1~dnorm(0,1)

pmb2~dnorm(0,1)

pmb3~dnorm(0,1)

d0~dnorm(0,.1)

d1~dnorm(0,.1)

xi2~dnorm(0,.1)

xi3~dnorm(0,.1)

xi4~dnorm(0,.1)

xi5~dnorm(0,.1)

taudelta1~dgamma(.1,.1)

taudelta2~dgamma(.1,.1)

taualpha1~dgamma(.1,.1)

taualpha2~dgamma(.1,.1)

}
