# Supplementary material for: A flexible Bayesian hierarchical model of preterm birth risk among US Hispanic subgroups in relation to maternal nativity and education
Source: BMC Med Res Methodol. 2011 Apr 19;11:51. doi: 10.1186/1471-2288-11-51 (PMC3108375; doi:10.1186/1471-2288-11-51)
Supplement: Additional file 2 — Appendix Figures. Appendix Figure A1. Age-adjusted risks of preterm birth (PTB) by nativity, education and Hispanic subgroup, women ≥20 years old, New York City, 1995-2003. Appendix Figure A2. Fully-adjusted risks of preterm birth (PTB) by nativity, education and Hispanic subgroup, women ≥20 years old, New York City, 1995-2003. Appendix Figure A3. Age-adjusted risk differences for the effect of nativity on preterm birth (PTB) by education and Hispanic subgroup, women ≥20 years old, New York City, 1995-2003. Appendix Figure A4. Fully-adjusted risk differences for the effect of nativity on preterm birth (PTB) by education and Hispanic subgroup, women ≥20 years old, New York City, 1995-2003. Appendix Figure A5. Comparison of age-adjusted to fully-adjusted risk differences for the effect of nativity on preterm birth (PTB) by education and Hispanic subgroup, women ≥20 years old, New York City, 1995-2003. Appendix Figure A6. Trace plots for several selected terms in the fully adjusted model (α10, δ10 and τ0) for native born Mexican-American women. [file 1471-2288-11-51-S2.DOC]

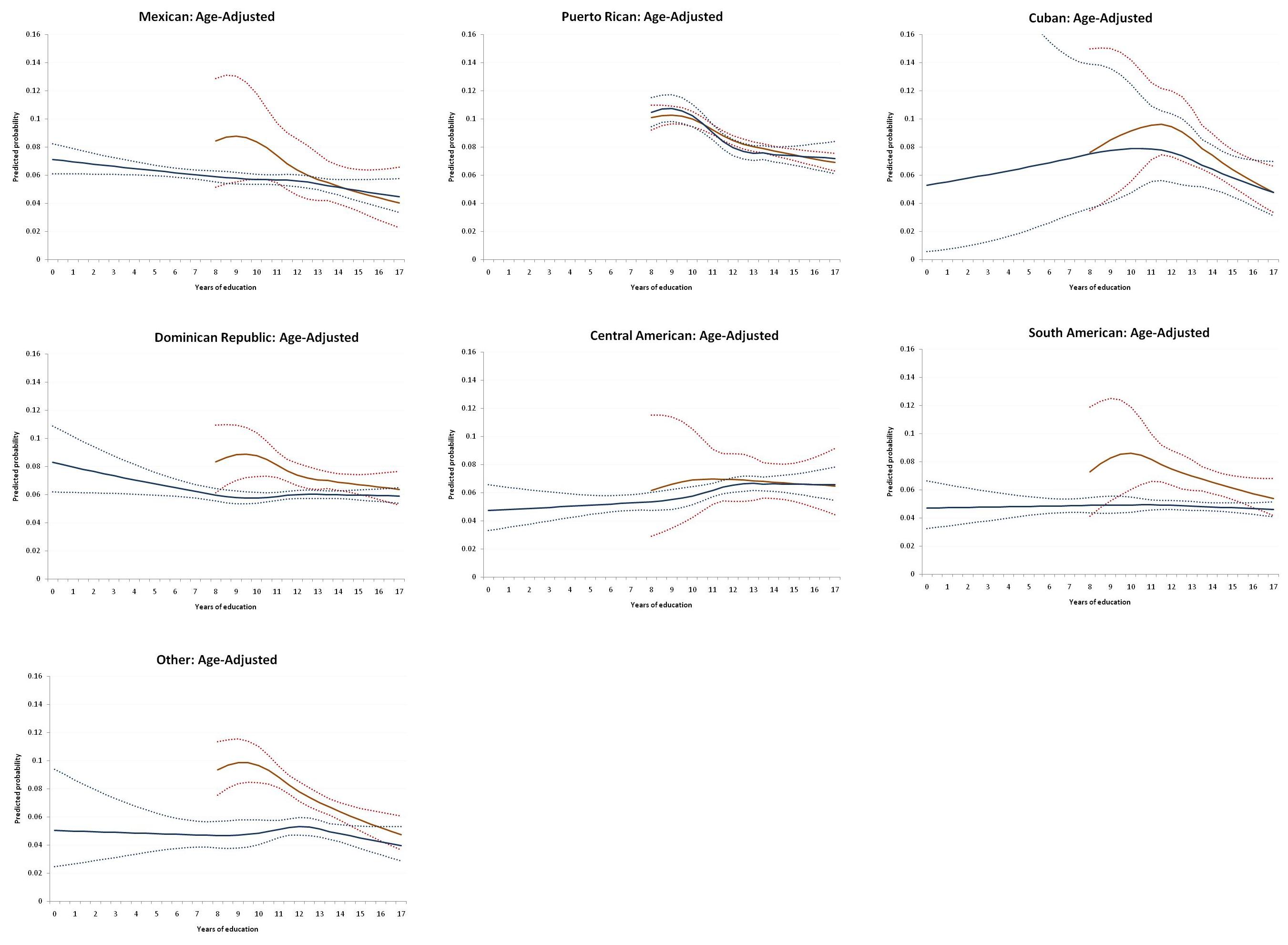


Appendix Figure A1. Age-adjusted risks of preterm birth (PTB) by maternal nativity

and education levels by Hispanic subgroups, for women ≥20 years old, New York City,

1995-2003. Blue line is foreign born and Orange line is native born. Solid lines are

point estimates and dotted lines are 95% posterior intervals as measures of precision.


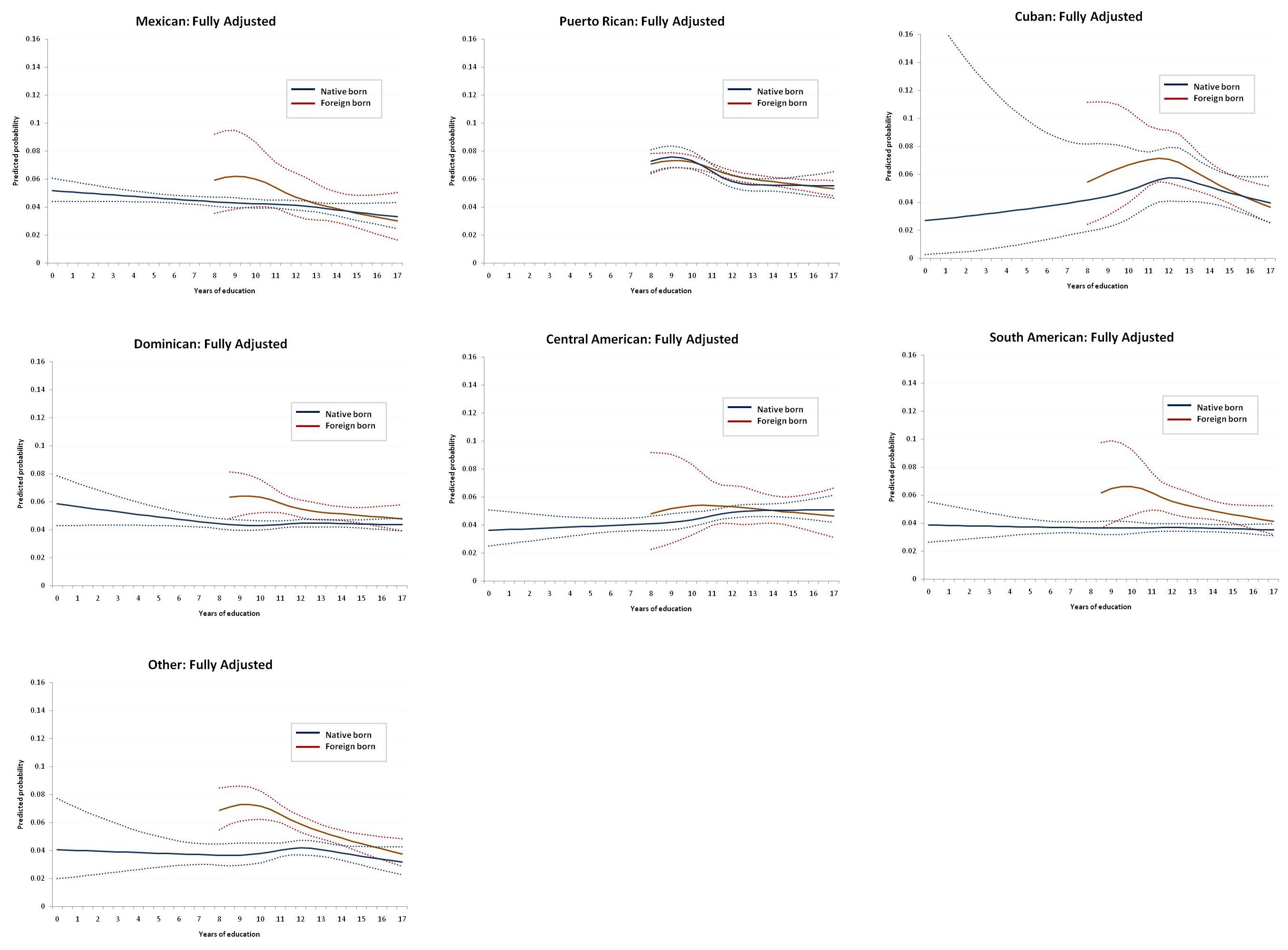


Appendix Figure A2. Fully-adjusted risks of preterm birth (PTB) by maternal nativity

and education levels by Hispanic subgroups, for women ≥20 years old, New York City,

1995-2003. Blue line is foreign born and Orange line is native born. Solid lines are

point estimates and dotted lines are 95% posterior intervals as measures of precision.


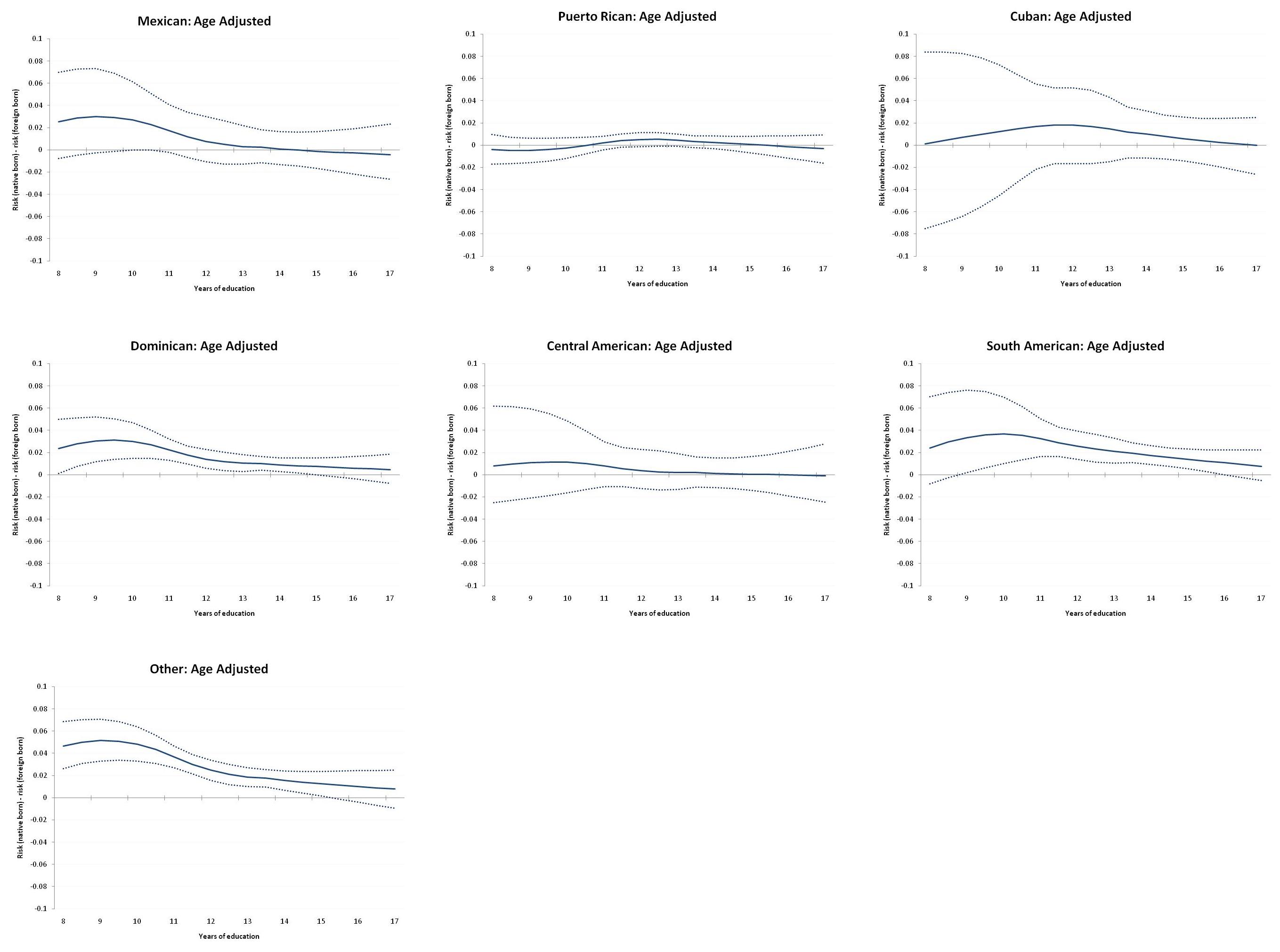


Appendix Figure A3. Age-adjusted risk differences for the effect of maternal nativity

on preterm birth (PTB) by education levels and Hispanic subgroups, for women ≥20

years old, New York City, 1995-2003. Solid lines are point estimates and dotted lines

are 95% posterior intervals as measures of precision.


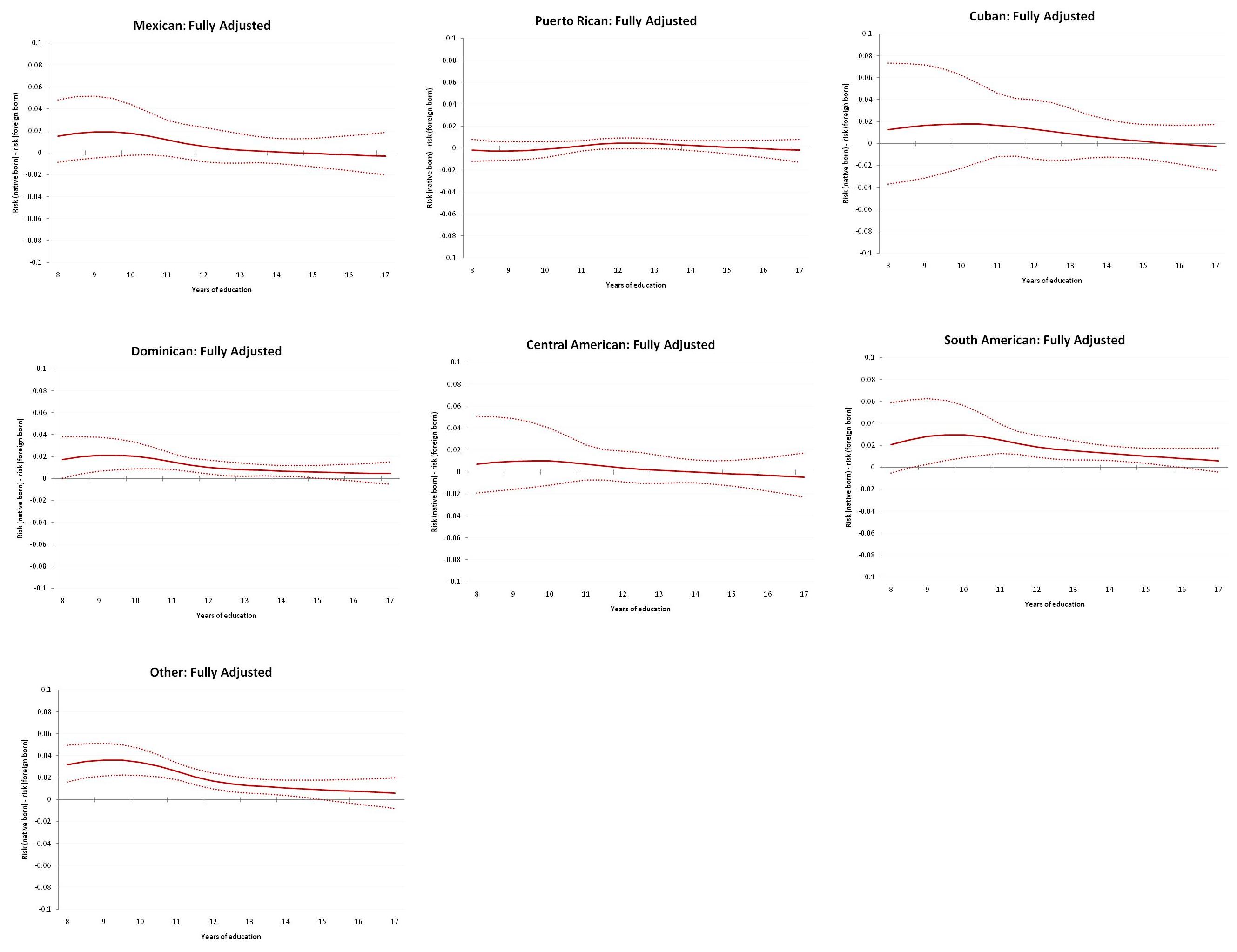


Appendix Figure A4. Fully-adjusted risk differences for the effect of maternal nativity

on preterm birth (PTB) by education levels and Hispanic subgroups, for women ≥20

years old, New York City, 1995-2003. Solid lines are point estimates and dotted lines

are 95% posterior intervals as measures of precision.


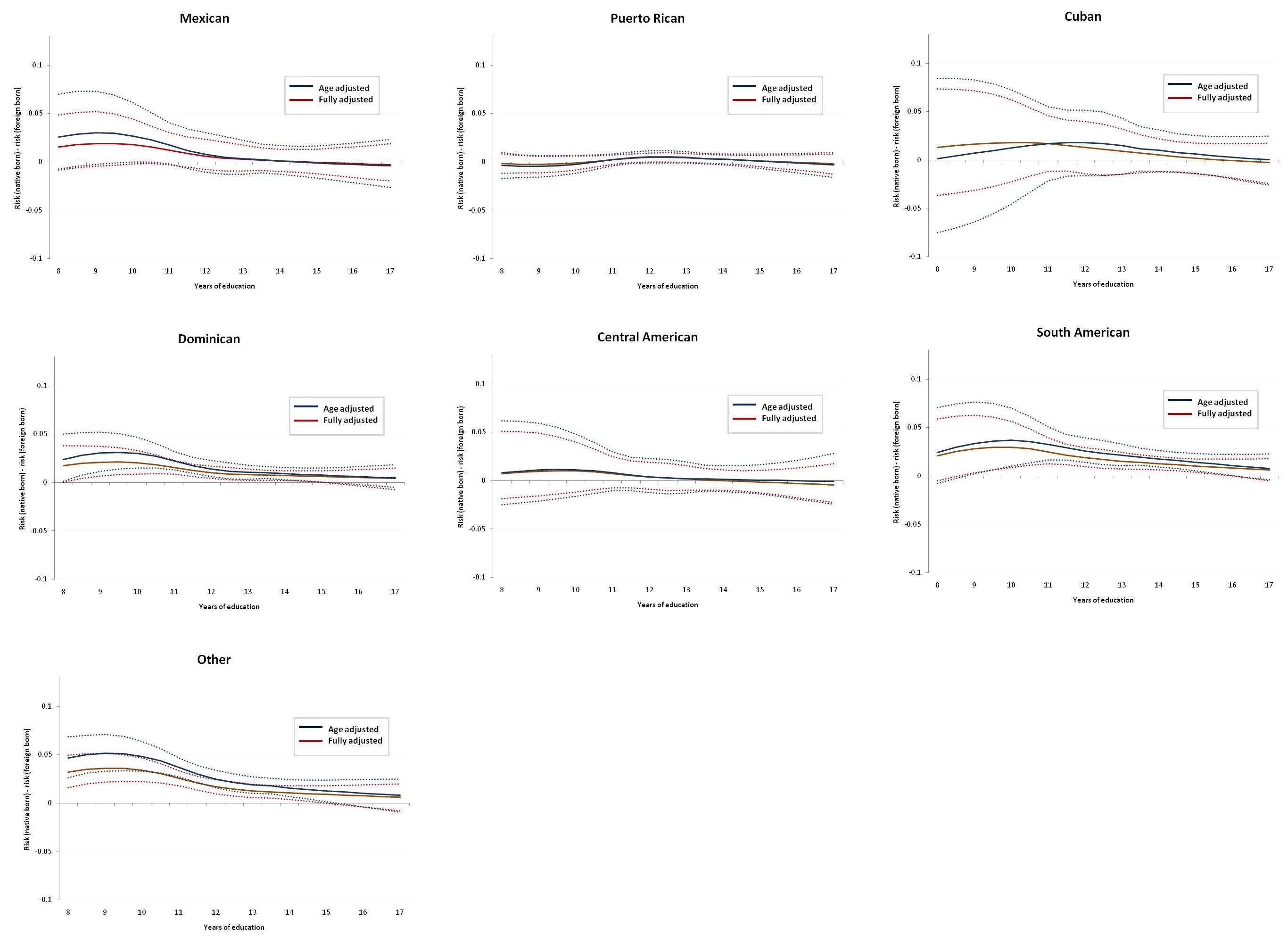


Appendix Figure A5. Comparison of age-adjusted to fully-adjusted risk differences for

the effect of maternal nativity on preterm birth (PTB) by education levels and Hispanic

subgroups, for women ≥20 years old, New York City, 1995-2003. Blue line is age-adjusted

and orange line is fully-adjusted. Solid lines are point estimates and dotted lines are 95%

posterior intervals as measures of precision.


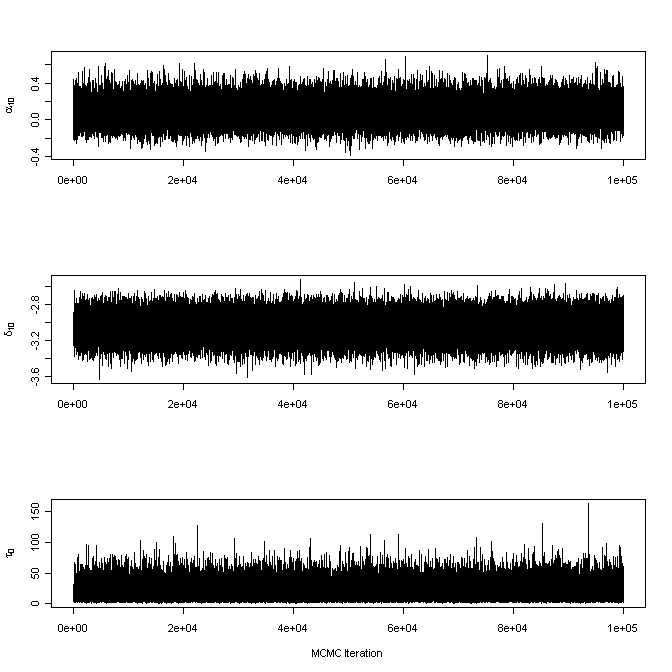


Appendix Figure A6.

Trace plots for several selected terms in the fully adjusted model (α_10_, δ_10_ and τ_0_). These plots are for native born Mexican-American women, which is one of the smaller groups, and they show good convergence properties for all three parameters. Good behavior in a small group is reassuring, since it is in the smaller groups where the precision term might be most likely to converge poorly.
